# Is a drainage time of less than 24 h sufficient after chronic subdural hematoma evacuation?

**DOI:** 10.1007/s00701-023-05511-y

**Published:** 2023-02-08

**Authors:** Andreas Bartley, Tobias Hallén, Magnus Tisell

**Affiliations:** 1 Department of Clinical Neuroscience, Institute of Neuroscience and Physiology, University of Gothenburg, Sahlgrenska Academy, Box 430, 40530 Gothenburg, Sweden; 2 Department of Neurosurgery, Sahlgrenska University Hospital, Blå Stråket 5, 41345 Gothenburg, Sweden

**Keywords:** Chronic subdural hematoma, Drainage, Duration, Time, Recurrence

## Abstract

**Background:**

It is well established that the use of a postoperative drain after chronic subdural hematoma surgery reduces recurrence rates, and it is common to use a postoperative drain for longer than 24 h. It is unclear whether this is superior to a shorter drainage time of less than 24 h. Our aim was to compare a postoperative drainage longer or shorter than 24 h after chronic subdural hematoma evacuation.

**Materials and methods:**

In this retrospective single centre study, 207 adult patients undergoing chronic subdural hematoma evacuation with a postoperative drainage longer (LDT-group) or shorter (SDT-group) than 24 h were compared regarding recurrence, mortality within 6 months and complications requiring hospital admission within 30 days. Length of hospital stay was also recorded. An active subgaleal drain was used. In addition to the retrospective cohort, we also studied the total volume drained per hour after cSDH surgery in a prospective cohort of 10 patients.

**Results:**

Recurrence occurred in 12/96 (12.5%) in the LDT-group and in 13/111 (11.7%) patients in the SDT-group (*p* = 0.15). There was no significant difference between groups regarding recurrence, complications or mortality. The prospective cohort showed that most of the drainage occurred within the first hours after surgery.

**Conclusion:**

Our data show that a postoperative drainage duration of less than 24 h does not lead to an increase in recurrence, complications or mortality compared to a drainage time of more than 24 h. A shorter drainage duration (< 24 h) after cSDH surgery facilitated earlier mobilisation and shorter hospital stay.

**Supplementary Information:**

The online version contains supplementary material available at 10.1007/s00701-023-05511-y.

## Introduction

A chronic subdural hematoma (cSDH) is a collection of old blood and degraded blood products located between the arachnoid and the dura mater. The hematoma may expand over time causing symptoms such as neurological deficits, headache, impaired level of consciousness and even death. The treatment is typically surgical evacuation via burr-hole craniostomy, and it is one of the most frequent procedures performed in neurosurgical practice. Nevertheless, recurrence requiring reoperation is common with reported recurrence rates of 5–21% [1]. Previous studies have convincingly shown that the use of a postoperative drain after cSDH evacuation significantly reduces recurrence as well as morbidity and mortality [4, 9]. However, the optimal duration of drainage after surgery remains unclear and evidence-based guidelines are lacking. When reviewing the scientific literature, it seems common to use a drain for at least 48 h [2, 4, 6, 7, 12]. Previous retrospective studies by Kale et al. and Yu et al. have even indicated that a drainage time of several days was beneficial to reduce recurrence after cSDH surgery [6, 11]. However, a recent randomised controlled trial (RCT) from Denmark compared drainage times of 24 versus 48 h and found no significant difference in recurrence or mortality [8].

From January 1, 2011, the postoperative routine was changed at our department from keeping an active drain for longer than 24 h to shorter than 24 h (often less than 12 h), and since 2011 we keep a drain for less than 24 after cSDH evacuation. Our hypothesis was that a drainage time of less than 24 h is sufficient after cSDH evacuation. Consequently, the aim of this retrospective single centre pilot study was to compare recurrence, complication frequency, length of hospital stay and mortality in patients with a postoperative drainage time of less than 24 h to patients with a drainage time longer than 24 h. An additional aim was to perform a prospective observational study of the total volume drained per hour, in a separate cohort of ten patients. If this prospective cohort would show that most of the drainage occurs during the first 24 h after surgery, it would support a drainage time of less than 24 h.

## Materials and methods

### Study design

The Department of Neurosurgery at the Sahlgrenska University Hostpital (Gothenburg, Sweden) has a geographically based catchment area of 1.9 million inhabitants in western Sweden. Approximately, 120–160 surgeries for cSDH are performed at the department yearly. In this retrospective single centre study, we compared the effect of drainage time (> 24 h vs. < 24 h) for all adult patients undergoing chronic subdural hematoma evacuation at our department. Patients with a minimum drainage time of 24 h or longer (long drainage time group, LDT) were recruited during January–September 2010, while patients with a drainage time shorter than 24 h (short drainage time group, SDT) were included during January–September 2011. The primary endpoint was recurrence requiring reoperation and secondary endpoints were mortality, length of hospital stay and complications requiring hospital admission within 30 days after surgery. Each patient had a follow-up of 6 months regarding recurrence and mortality.

As a complement to our retrospective study, we also performed a prospective observational study of drainage volume in a separate cohort of 10 consecutive patients undergoing cSDH evacuation during November–December 2021. After cSDH surgery, the amount of drainage was measured every hour and recorded until drain removal. The drain was removed after a period of at least 4 h without any observed drainage. This was in accordance with our normal clinical praxis. To make certain any decline in drainage was not caused by reduced suction, the active suction of the subgaleal drain was also checked hourly.

Both the retrospective and prospective cohorts shared the same criteria for inclusion and exclusion in the study. Inclusion criteria were all patients older than 18 years undergoing burr-hole evacuation of cSDH combined with an active subgaleal drain. Exclusion criteria were surgery for cSDH by other means than burr-hole evacuation or if a drain was not used.

### Ethical approval

The Swedish Ethical Review Authority concluded that the study did not need a formal ethical approval and found no ethical concerns regarding the study (DNR 2021–00,048). This applied to both the retrospective and the prospective cohorts studied.

### Surgical technique

The hematoma was evacuated via 1–2 burrholes, the subdural space was irrigated and followed by insertion of an active subgaleal drain (Abdovac FG 10 with troacar, 25 mm Hg, Wellspect Healthcare) as described by Gazzeri et al. [3]. All surgeons at our department employed the same surgical technique. The patient was kept in the supine position until drain removal. A routine postoperative computed tomography (CT) was not performed unless the patient failed to show clinical improvement after surgery. After drain removal, most patients could be discharged to their local hospital the day after surgery. If the patient developed recurrent symptoms during follow-up, a CT would be performed, and our department contacted subsequently. The only difference in surgical technique between the patients operated in 2010 and 2011 were drainage times longer than 24 h or shorter than 24 h respectively. The same surgical technique was also used for the prospective observational cohort investigated in 2021.

### Statistical analysis

Analysis of the primary endpoint was performed with *X*^2^-test for frequency comparison. Secondary endpoints were similarly analysed with *X*^2^-test for categorical data. Student’s *t*-test was used for normally distributed numerical data and Mann–Whitney *U*-test was used if skewed. Statistical significance was set at 5%. IBM SPSS Statistics (version 25) and Microsoft Excel (version 2202, 14,931.20660) was used for statistical analyses.

## Results

### Retrospective cohort

**Fig. 1:**
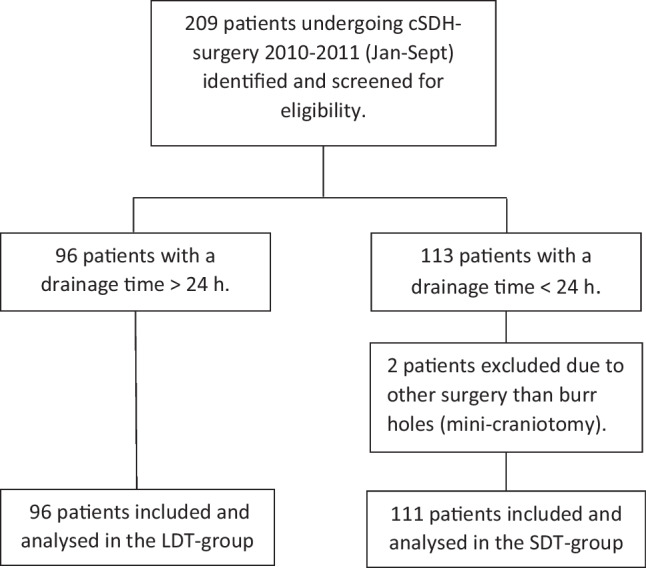
Study flowchart

A total of 96 patients in the LDT-group and 111 in the SDT-groups had a complete follow-up of 6 months. Two patients in the SDT-group were excluded due to other cSDH surgery than burr-hole craniostomy (Fig. 1). None of the excluded patients had a recurrence within 6 months. There were no significant differences between the SDT- and the LDT-group regarding demographics or other known risk factors of cSDH recurrence (Table 1*)*.

**Table 1:** Characteristics of the patient groups treated with different drainage times after evacuation of chronic subdural hematoma. Data presented as mean (SD) or *n* (%). cSDH, chronic subdural hematoma; LDT, long drainage time (> 24 h); SDT, short drainage time (< 24 h)

Variable	LDT-group *N* = 96	SDT-group *N* = 111	*p*-value
Age (years)	75.6 (10.0)	75.3 (11.2)	0.84
Women	32 (33.3%)	34 (30.1%)	0.10
Duration of surgery (minutes)	43.9 (9.6)	42.3 (5.4)	0.13
Antithrombotic medication	40 (41.7%)	48 (43.2%)	0.12
Maximal hematoma width (mm)	20.1 (4.9)	21.4 (4.1)	0.54
Bilateral hematoma	21 (21.8%)	22 (19.8%)	0.10
Midline shift (mm)	8.0 (4.8)	9.1 (4.5)	0.09
Membranous cSDH	12 (12.5%)	17 (15.3%)	0.07
Separated/layered cSDH	5 (5%)	8 (7%)	0.11

Mean (range) drainage time in the SDT-group was 12.0 h (7–20) and in the LDT-group 24.8 h (24–32), thereby demonstrating a significantly shorter overall drainage time in the SDT-group and the relevance of comparing the two groups (*p* < 0.001). In the LDT-group, recurrence occurred in 12 of 96 patients (12.5%) compared to recurrence in 13 of 111 patients (11.7%) in the SDT-group during the six month follow-up. The difference in recurrence between the groups was not statistically significant *(p *= 0.15; OR 0.92 [95% CI 0.5–1.7]).

Mortality in the LDT-group was 6 of 96 (6.3%) and 4 of 111 (3.6%) patients for the SDT-group. The difference in mortality between groups was not statistically significant (*p* = 0.10). In total, there were 4 cases (4.2%) in the LDT-group (one case each of acute SDH, postoperative seizure, pneumonia, and sepsis of unknown cause) and 5 cases (4.5%) in the SDT-group (one case each of acute myocardial infarction, urinary tract infection, pneumonia, atrial fibrillation and heart failure) that developed complications requiring hospital admission within 30 days (*p* = 0.15). Mean length of hospital stay (at the neurosurgical department) was 3.6 days for the LDT-group compared to 2.7 days for the SDT-group (*p* = 0.01).

### Prospective cohort

**Fig. 2:**
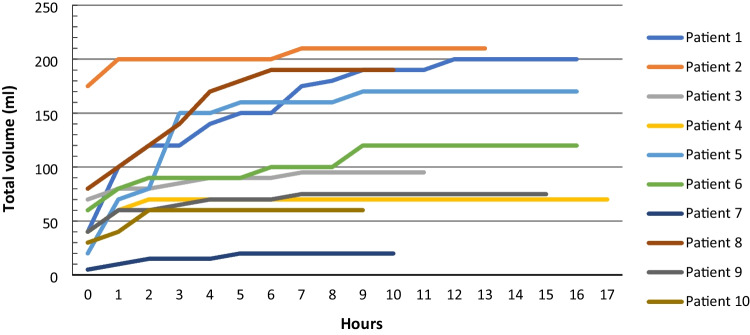
Total volume drained after chronic subdural hematoma (cSDH) evacuation in a prospective cohort of 10 patients. Time point 0 indicates arrival from the operating room to the neurosurgical observation unit (within 1 h after surgery). Termination of lines indicate drain removal. The observations show that most of the drainage occurred within the first five hours after surgery

The prospective observational cohort of ten consecutive patients undergoing cSDH evacuation had a mean (range) drainage time of 13.3 h (9–17) and a mean drained volume of 121 ml (20–210). The total volume drained from each patient until drain removal is shown in Fig. 2. Mean (range) age was 81 years (64–91) and 20% were women. Detailed characteristics for each patient in the prospective cohort can be found as Supplementary material (Appendix 1). These findings revealed that 5 to 6 h after surgery, 8 of 10 (80%) patients had drained 90–95% of their total volume. The remaining two patients had drained more than 95% of their total volume after 9 h. Recurrence was seen in one patient with a drainage time of 16 h and a total drained volume of 170 ml. No cases of mortality or complications were registered in the prospective cohort within 6 months after surgery.

## Discussion

The aim of our pilot study was to explore if a postoperative drainage time of less than 24 h is sufficient regarding recurrence rate, complication frequency and mortality compared to a longer drainage time. Our data show that a postoperative drainage time of less than 24 h (SDT-group) does not increase the recurrence rate, complication frequency or mortality in comparison to a drainage time longer than 24 h (LDT-group). This in turn facilitated earlier patient mobilisation and optimisation of hospital resources due to a significantly shorter hospital stay. A review of the literature revealed that keeping a drain for at least 24–48 h combined with bedrest seems common [2, 4, 6, 7, 12]. Our results contradict earlier retrospective studies concluding that a drainage time of at least 3–4 days is indicated after cSDH evacuation [6, 11]. In support of shorter drainage times, a RCT from 2022 showed that there was no difference in recurrence or mortality between a drainage time of 24 compared with 48 h. However, the authors found that drain production was significantly higher after 48 h compared to 24 h with mean values of 146 and 88 ml respectively [8]. In comparison, our prospective cohort had a mean drain production of 121 ml with a mean drainage time of 13.3 h. The difference may be explained by the drain used. We use an active subgaleal drain as opposed to the mentioned studies above who all used a passive subdural drain [6, 8, 12]. It is possible that an active subgaleal drain evacuates residual cSDH more effectively than a passive subdural drain. In a Scandinavian multicentre study comparing passive subdural and active subgaleal drains, the latter was recommended due to the best ratio between recurrence and complications [10]. A RCT-study by Soleman et al. came to the same conclusion with higher rates of iatrogenic brain injury and infection when using a passive subdural drain compared to an active subgalaeal drain [11].

To support our retrospective data, we studied a prospective cohort of 10 patients regarding the actual drainage after surgery. The results showed that most of the drainage occurred during the first hours postoperatively, further indicating that a drainage time of less than 24 h is sufficient.

Limitations of the study include the single centre retrospective design and that the retrospective data are from 2010 to 2011 when the routine of drainage time was changed at our department (from > 24 h to < 24 h). However, we believe that the comparison of the 2010 and 2011 cohorts is justified because we have since continued to use a drainage time of less than 24 h. Furthermore, it cannot be excluded that individual surgeons change their surgical behaviour when a routine is changed, or a new intervention is introduced. To address this matter, we compared the duration of surgery which did not differ between groups, indicating that the surgery performed was unchanged.

Strengths of the study are that the patients included all come from a defined geographical area were all cases of cSDH recurrence are treated at our department. Furthermore, digitalised medical records used at our department as well as the local hospitals in our catchment area enabled a complete follow-up of 6 months regarding all the endpoints. Finally, all surgeons at our department used the same surgical technique when evacuating cSDH.

To further investigate the results of this pilot study an RCT-study comparing drainage times and its impact on recurrence, mortality and complications is needed. Interestingly, a Danish nationwide RCT-study comparing drainage times of 6, 12 or 24 h is planned [5]. However, since the planned RCT-study explores drainage times with a passive subdural drain a separate RCT-study exploring drainage times with an active subgaleal drain might also be of interest.

## Conclusion

Keeping an active subgaleal drain for less than 24 h after cSDH surgery did not lead to an increase in recurrence, mortality or complications as compared to a drainage time longer than 24 h. A drainage time less than 24 h after surgery did facilitate earlier patient mobilisation and a shortened hospital stay.

## Supplementary Information

Below is the link to the electronic supplementary material.

Supplementary file1 (DOCX 20 KB)

## Data Availability

The data that support the findings of this study are available on request from the corresponding author, Dr Andreas Bartley.
