# Supplementary material for: Is a drainage time of less than 24 h sufficient after chronic subdural hematoma evacuation?
Source: Acta Neurochir (Wien). 2023 Feb 8;165(3):711–5. doi: 10.1007/s00701-023-05511-y (PMC10006057; doi:10.1007/s00701-023-05511-y)
Supplement: Supplementary file 1 — Supplementary file1 (DOCX 20 KB) [file 701_2023_5511_MOESM1_ESM.docx]

**Appendix 1.**

*Detailed characteristics of the ten patients in the prospective cohort of the study.*
